# Protective immunity against *Trichinella spiralis* in mice elicited by oral vaccination with attenuated *Salmonella*-delivered TsSP1.2 DNA

**DOI:** 10.1186/s13567-018-0582-2

**Published:** 2018-09-06

**Authors:** Jie Feng Li, Kai Xia Guo, Xin Qi, Jun Jun Lei, Yue Han, Shu Wei Yan, Peng Jiang, Chuan Yu, Xiang Chao Cheng, Zhong Quan Wang, Jing Cui

**Affiliations:** 1 Department of Parasitology, Medical College, Zhengzhou University, Zhengzhou, 450052 China; 2 Key Lab of Animal Disease and Public Health, College of Animal Science and Technology, Henan University of Science and Technology, Luoyang, 471003 China

## Abstract

Trichinellosis is a worldwide important food-borne zoonosis caused mainly by ingesting raw or undercooked pork infected with *Trichinella spiralis* larvae. The development of vaccine is needed for preventing swine from *Trichinella* infection to ensure pork safety. Previous studies showed that *T. spiralis* serine protease 1.2 (TsSP1.2) is a vaccine candidate against *Trichinella* infection. In this study, the complete TsSP1.2 cDNA sequences were cloned into pcDNA3.1, and the rTsSP1.2 DNA was transformed into attenuated *Salmonella typhimurium* strain ΔcyaSL1344. Oral vaccination of mice with *Salmonella*-delivered rTsSP1.2 DNA vaccine induced an obvious intestinal mucosal IgA response and a systemic Th1/Th2 immune response; the vaccinated mice showed a 33.45% reduction of intestinal adult worms and 71.84% reduction of muscle larvae after *T. spiralis* larval challenge. The protection might be due to the rTsSP1.2-induced production of specific anti-TsSP1.2 sIgA, IgG, IgG1/IgG2a, and secretion of IFN-γ, IL-4 and IL-10, which protected intestinal mucosa from the parasite invasion, inhibited worm development and reduced female fecundity. The results indicate that the attenuated *Salmonella*-delivered rTsSP1.2 DNA vaccine offers a prospective strategy for the prevention and control of animal *Trichinella* infection.

**Electronic supplementary material:**

The online version of this article (10.1186/s13567-018-0582-2) contains supplementary material, which is available to authorized users.

## Introduction

Trichinellosis, an important food-borne zoonosis worldwide, is principally caused by the parasitic nematode *Trichinella spiralis* [1]. *T. spiralis* infection is acquired mainly from ingesting raw or semi-cooked meat contained with the encapsulated infective muscle larvae (ML). Domestic pork is a major source of this infection in southern America, Southeast Asia, China and other countries [2–5]. In China, 12 trichinellosis outbreaks caused by eating contaminated pork were recorded from 2004 to 2009 [6]. *Trichinella* infection in domestic pigs has a serious impact on meat safety and public health [7]. Because a large amount of pork was consumed in the world, the development of vaccine is needed for preventing swine from *Trichinella* infection to ensure pork safety [8–10].

After infected meat is ingested, ML are liberated from the capsule in the host’s stomach, where they develop into intestinal infective larvae (IIL) in the small intestine, before invading the intestinal mucosa where they develop into adult worms (AW) after undergoing four molts [11]. The AW dwell in the small intestine with their head burrowing into the intestinal epithelium, and ovoviviparous female adults deposit the newborn larvae (NBL) which penetrate into intestinal mucosa and migrate to skeletal muscles, where they become the encapsulated ML. The IIL invasion of host intestinal mucosa is the key step in the process of this infection. The intestinal mucosa is the first natural barrier to resist *Trichinella* invasion and the primary interaction location for the intestinal parasitic nematode and host [12]. Hence, it is important to elicit local intestinal mucosal immune response for immune protection against intestinal *Trichinella* infection [13].

In our previous studies, a *T. spiralis* serine protease 1.2 (TsSP1.2) (GenBank Accession No. EU302800) was identified in excretory–secretory (ES) and surface proteins of *T. spiralis* ML and IIL by immunoproteomics [14–17]. The bioinformatics analysis demonstrated that the complete cDNA sequences of TsSP1.2 gene are 1137 bp. The TsSP1.2 open reading frame (ORF) encodes a 35.5 kDa protein of 313 amino acids. The signal peptide is located between 1 and 27aa. The TsSP1.2 has a domain of trypsin-like serine protease. The TsSP1.2 gene was cloned and expressed in our laboratory. Partial inhibition of larval invasion of intestinal epithelial cells (IEC) was observed using anti-TsSP1.2 serum; immunization of mice with rTsSP1.2 producing a partial immune protection against *T. spiralis* infection [18]. In the present study, a DNA vaccine targeting TsSP1.2 was delivered by attenuated *Salmonella typhimurium* strain ΔcyaSL1344. Local mucosal and systemic immune responses were investigated in mice vaccinated orally with *Salmonella*-delivered TsSP1.2 DNA vaccine, and protection against larval challenge was also evaluated.

## Materials and methods

### Mice and parasite

Female 5-week old BALB/c mice were provided by the Experimental Animal Center of Zhengzhou University (Zhengzhou, China). These mice were bred in individual ventilated cages (IVC, Suzhou Fengshi Laboratory Animal Equipment Co., Ltd, Suzhou, China). *T. spiralis* strain (ISS534) was obtained from a domestic pig in central China. We kept this strain by passages in BALB/c mice every 6 months.

### Collection of various worm stages

The ML were acquired from experimentally *T. spiralis*-infected mouse carcass at 42 days post-infection (dpi) using an artificial digestion method [19, 20]. The IIL were harvested from infected mouse intestines at 6 hours post-infection (hpi) [15], and the adult worms (AW) were respectively separated from intestines at 3 and 6 dpi [21]. The newborn larvae (NBL) were collected from the 6 dpi female adults which were cultured in RPMI-1640 media for 24 h at 37 °C [22].

### Attenuated *Salmonella* strain

The attenuated *S. typhimurium* ΔcyaSL1344 strain in which the cya gene was deleted was prepared by the Animal Disease and Public Health Key Laboratory, Henan University of Science and Technology. The attenuated *Salmonella* strain was grown in Luria–Bertani (LB) broth containing 2% NaCl [10] and applied as a vector of the expression plasmid pcDNA3.1 carrying TsSP1.2 gene.

### Plasmid construction and transformation

The complete TsSP1.2 cDNA sequence was amplified by PCR using the following primers: 5′-T**AAGCTT**GCCACCATGAAACGCTGGCAC-3′, 5′-CTT**CTCGAG**TTAGCCGGCAT GCAGCAGT-3′. The *Hind*III and *Xho*I sites are bold. The amplified DNA fragment were cloned into pcDNA3.1 (Invitrogen, Carlsbad, USA). The insert sequence and ORF was verified by two directional DNA sequencing. The recombinant pcDNA3.1-TsSP1.2 and the control empty plasmid pcDNA3.1 were electroporated into the bacteria [10]. The transformant was selected on LB agar with 50 μg/mL ampicillin and identified by PCR amplification with restriction enzyme digestion and DNA sequencing (GENEWIZ, Suzhou, China).

### Preparation of recombinant TsSP1.2

The rTsSP1.2 protein was expressed in an *Escherichia coli* BL21 (DE3) and identified in our laboratory [18]. The purified rTsSP1.2 had a molecular weight of 78.5 kDa and consisted of a 35.5 kDa TsSP1.2 protein and a maltose-binding protein tag (43 kDa). An amylose pre-packed column (NEB Ltd, China) was utilized for purification of rTsSP1.2 [23].

### RT-PCR and immunofluorescent test (IFT) for detecting the in vitro transcription and expression of TsSP1.2

Baby hamster kidney cell 21 (BHK-21) were cultured in plates with DMEM media supplemented with 100 U/mL penicillin, 100 μg/mL streptomycin and 10% inactivated fetal bovine serum (FBS) at 37 °C in 5% CO_2_. When the BHK 21 cells were grown to 90% confluence, the cells were collected by trypsinization and transfected with pcDNA3.1-TsSP1.2 with a cationic lipid Lipofectamine 2000 (Invitrogen, USA) at the ratio of 0.8 μg DNA: 2 μL lipid per well in serum-free DMEM media at 37 °C for 48 h. Total RNA was extracted from the BHK-21 cells 24 h after transfection, and the transcription levels of TsSP1.2 mRNA in transfected cells were assayed by RT-PCR with TsSP1.2-specific primers as listed above [24]. Expression of TsSP1.2 protein in cells was observed using IFT [25]. Briefly, BHK-21 cells were cultivated as monolayers and fixed with cold acetone for 15 min. After being washed with PBS, the cells were blocked with 5% normal goat serum at 37 °C for 1 h, and then probed with 1:10 dilutions of mouse anti-rTsSP1.2 sera at 4 °C overnight. Then, the cells were incubated with a 1:100 dilution of anti-mouse IgG-FITC conjugate (Santa Cruz Biotechnology, Dallas, Texas, USA) for 1 h at room temperature. The cells were stained by 0.01% Evans blue for eliminating non-specific staining. Finally, the cells were observed and photographed by fluorescence microscopy (Olympus, Japan).

### Immunization design and sample collection

One hundred and twenty mice were divided into 3 groups (40 mice per group). On day 0, the immune group of mice was infused orally with 1 × 10^8^ cells of Δ*cya* SL1344/pcDNA3.1-TsSP1.2. The empty Δ*cya*SL1344/pcDNA3.1 or PBS was given orally to control mice. All mice were boosted two times at a 10-day interval. Before oral inoculation, all mice were infused by gavage with 100 μL of 10% NaHCO3 for neutralizing stomach acidity [26]. At days 0, 10, 20 and 30 after immunization, blood samples were collected from 10 mice of each group. Five mice from each group were euthanized, and small intestines, spleens and mesenteric lymph nodes (MLN) were taken to assay the immune responses.

### RT-PCR and IFT for detecting the in vivo transcription and expression of TsSP1.2

At 1-week post last immunization, TsSP1.2 mRNA was detected in spleens and MLN of vaccinated mice by RT-PCR with TsSP1.2-specific primers as mentioned above. Total RNA of spleens and MLN was prepared using TRIzol (Invitrogen, Carlsbad, USA). Mouse β-actin of spleens and MLN was also amplified as an internal control [27]. PCR product was electrophoresed with 1% agarose gels. To determine the TsSP1.2 expression in vivo, the spleens and MLN of mice vaccinated with TsSP1.2 DNA vaccine were fixed and microtomed with thickness of 5 μm. The IFT was performed as described above [25]. The only difference was that the frozen section was not stained with Evans blue.

### Determination of anti-rTsSP1.2 IgG and its subclass by ELISA

Specific anti-rTsSP1.2 antibodies (total IgG, IgG1 and IgG2a) in serum of vaccinated mice were measured by standard indirect ELISA at 10 days following each vaccination [28, 29]. Microtiter plate was coated with rTsSP1.2 (2 μg/mL) at 4 °C overnight. After being washed, 5% skim milk was utilized for blockage at 37 °C for 2 h, and incubated with 1:100 dilutions of mouse sera. After being washed again, the plate was incubated with HRP-conjugated anti-mouse IgG, IgG1, or IgG2a (1:5000; Sigma-Aldrich). The coloration was developed by incubation with *o*-phenylenediamine dihydrochloride (OPD; Sigma) plus 30% H_2_O_2_. The absorbance at 490 nm was determined by a microplate reader (Tecan, Schweiz, AG, Switzerland).

### Determination of total and TsSP1.2-specific secretory IgA

To determine total and TsSP1.2-specific secretory IgA (sIgA) response, the intestinal washes were prepared [30]. Briefly, small intestine was cut into 10 cm long for each vaccinated mouse, and the intestinal interior was douched three times with 1 mL of cold PBS. After intestinal washing, the intestinal contents were centrifuged at 7300* g* for 10 min and the supernatant was collected. Intestinal secretory total IgA was determined by sandwich ELISA as described [10]. TsSP1.2-specific IgA was assayed by indirect ELISA using rTsSP1.2 (2 μg/mL) as the coating antigen.

### Recognition of native TsSP1.2 at different *T. spiralis* phases by IFT

Native TsSP1.2 on the surface and internal structures of various *T. spiralis* phases was observed by IFT with anti-TsSP1.2 serum and TsSP1.2-specific sIgA in intestinal washes from vaccinated mice [31, 32]. The intact worm or 2-μm sections of different stages was fixed in cold acetone, anti-TsSP1.2 serum or intestinal washes from vaccinated mice were used as primary antibody, and anti-mouse IgG- or IgA-FITC conjugate (1:100 dilution, Abcam, UK) served as the secondary antibody. After incubation and washing, intact worms and sections were observed by fluorescent microscopy (Olympus, Japan) [33, 34].

### Cytokine assays

The cells of spleen and MLN were harvested from immunized and non-immunized mice on days 0, 10, 20 and 30 after vaccination [10, 33]. Cell suspensions were prepared and their density was diluted to 2 × 10^6^ cells/mL in complete RPMI-1640 containing 10% FBS. Cell suspensions were cultured and stimulated with the rTsSP1.2 (4 μg/mL) in a humidified 5% CO_2_ atmosphere for 72 h at 37 °C. Non-stimulated cells were used as a negative control. Cytokines (IFN-γ, IL-4, and IL-10) in supernatant were assayed by ELISA and cytokine concentration was shown as pg/mL [35].

### Immune protection against challenge infection with *T. spiralis*

To assess the immune protection, 20 mice from each group were challenged orally with 300 *T. spiralis* ML 7 days after the third vaccination, euthanized at 7 dpi, and adult worms of small intestine were recovered and numerated [32]. The length of female adults was measured under light microscopy. The fecundity of adult females was determined after being incubated in 1640 medium for 72 h at 37 °C, and the NBL born by each female was numerated [36]. The muscle larval burden of the remaining mice were examined by artificial digestion method at 35 dpi [37]. Immune protection was evaluated as the worm burden reduction of intestinal adults and muscle larvae collected from the group vaccinated with ΔcyaSL1344/pcDNA3.1-TsSP1.2 with respect to those from the control mice that received only PBS [38, 39].

### Statistical analysis

All the data was analyzed with SPSS version 17.0 software. The data were shown as the mean ± standard deviation. The intra- and intergroup statistical analysis were performed with one-way ANOVA (LSD test). *P *< 0.05 was regarded as statistically significant.

## Results

### Construction of recombinant pcDNA3.1-TsSP1.2

The complete TsSP1.2 cDNA was cloned into pcDNA3.1. After being digested with* Hind*III and XhoI, an approximately 948 bp insert was observed in recombinant pcDNA3.1-TsSP1.2. Sequencing results revealed that the amplified TsSP1.2 fragment consisted of 948 bp, the predicted ORF encoded a protein of 315 amino acids of 35.2 kDa, with 99.58% identity to those of TsSP1.2 in GenBank (EU302800).

### The in vitro transcription and expression of TsSP1.2

**Figure 1:**
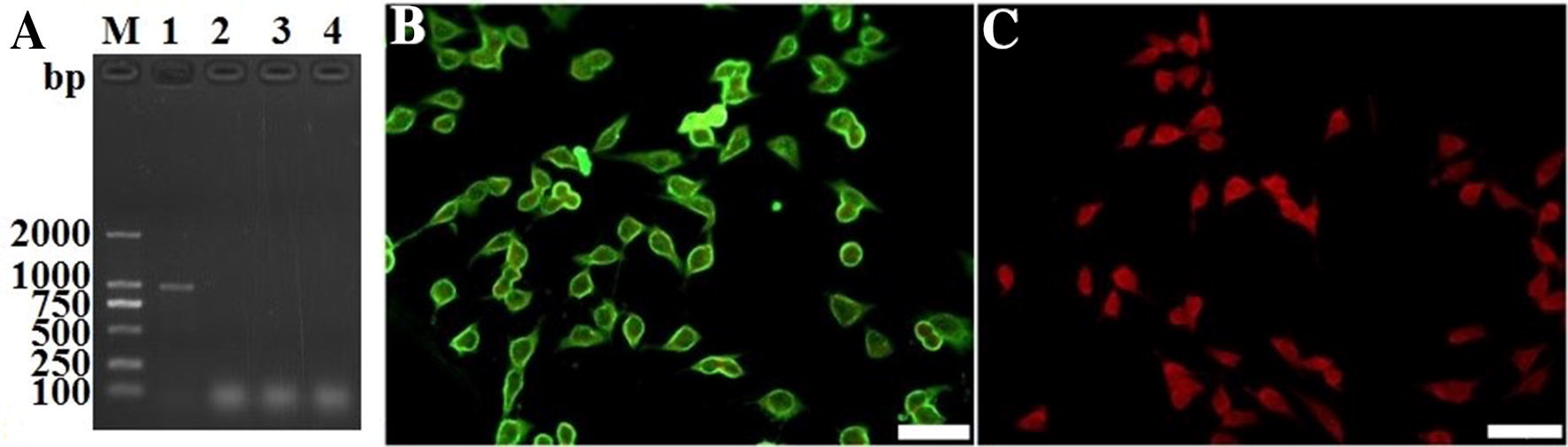
**The in vitro transcription and expression of TsSP1.2 in transfected BHK-21 cells. A** TsSP1.2 mRNA transcription in BHK-21 cells was detected by RT-PCR. l. M: DL2000 marker; 1: pcDNA3.1-TsSP1.2 transfected cells; 2: empty pcDNA3.1 transfected cells; 3: non-transfected normal cells; 4: Lipofectamine 2000 control. **B** TsSP1.2 expression in BHK-21 cells transfected with pcDNA3.1-TsSP1.2 was observed by IFT with anti-rTsSP1.2 serum. **C** The pcDNA3.1-transfected BHK-21 cells were used as negative control. The scale bar is 50 μm.

TsSP1.2 mRNA transcription in BHK-21 cells was assayed using RT-PCR. An amplified TsSP1.2 fragment was detected in cells transfected with pcDNA3.1-TsSP1.2, but not in cells transfected with empty pcDNA3.1 and non-transfected BHK-21 cells. TsSP1.2 protein expression in BHK-21 cells was observed by IFT using anti-rTsSP1.2 serum, intense immunostaining was detected in transfected cells, but not in non-transfected cells (Figure 1).

### The in vivo transcription and expression of TsSP1.2

**Figure 2:**
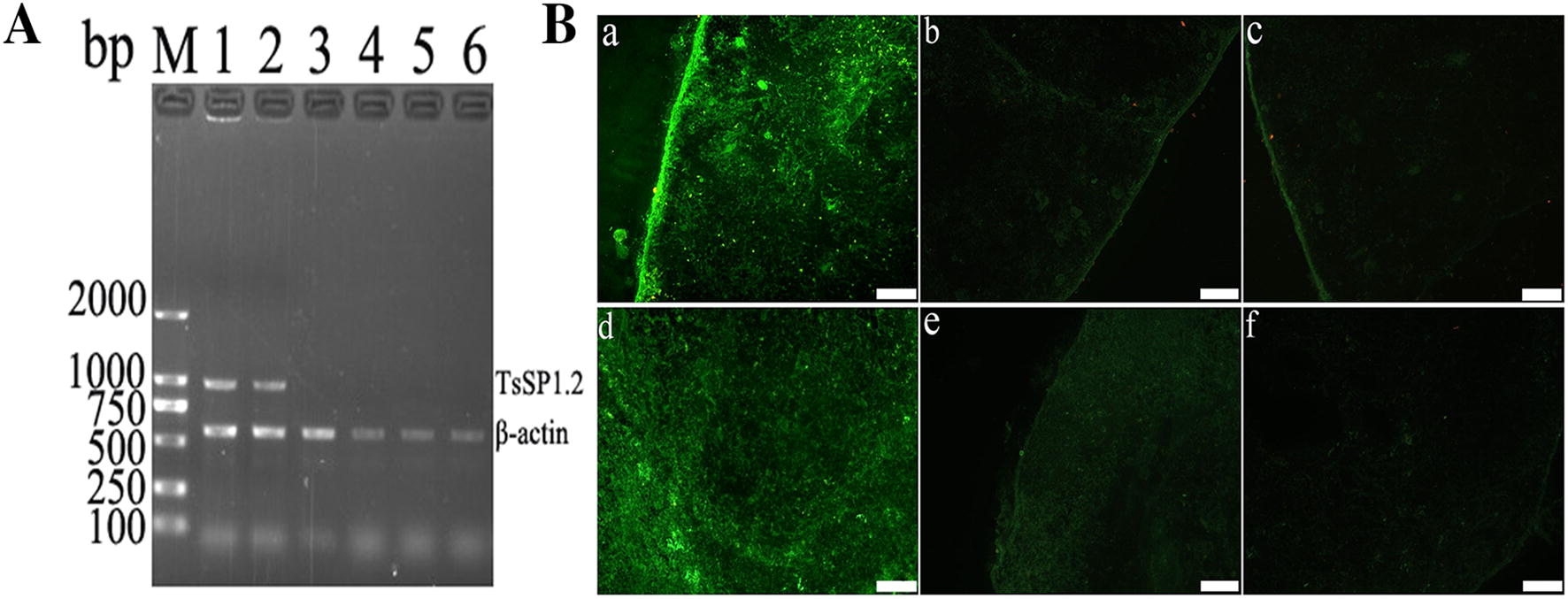
**The in vivo transcription and expression of TsSP1.2 in mice vaccinated with TsSP1.2 DNA/*S. typhimurium.*** **A** RT-PCR analysis of TsSP1.2 mRNA transcription. TsSP1.2 mRNA was detected in spleen (lane 1) and MLN (lane 2) of TsSP1.2-immunized mice, either in spleen (lane 3) or in MLN (lane 4) of mice vaccinated with only empty pcDNA3.1, not in spleen (lane 5) and MLN (lane 6) of mice inoculated with only PBS. **B** IFT detection of TsSP1.2 expression. The expression of TsSP1.2 was detected in spleen (a) and MLN (d) of TsSP1.2-immunized mice by IFT with anti-rTsSP1.2 serum, but not in spleen (c) and MLN (f) by IFT with pre-immune serum. By using anti-rTsSP1.2 serum, no distinct staining was observed in spleen (b) and MLN (e) of mice inoculated with only empty pcDNA3.1. Scale bar: 100 μm.

To determine the transcription of TsSP1.2 in the spleen and MLN tissues of vaccinated mice, total RNA was extracted from spleen and MLN, TsSP1.2 mRNA transcription was detected by RT-PCR in mice vaccinated with TsSP1.2 DNA vaccine, but not in mice inoculated with only empty pcDNA3.1. Murine β-actin cDNA was also amplified as an internal control. Immunostaining was detected in spleen and MLN tissue sections of mice vaccinated with TsSP1.2 DNA vaccine using IFT with anti-rTsSP1.2 serum but not in mice inoculated with only empty pcDNA3.1. When pre-immune serum was used, no evident staining was seen in tissue sections of vaccinated mice (Figure 2). The results revealed that TsSP1.2 was transcribed and expressed in mice vaccinated with Δ*cya*SL1344/pcDNA3.1-TsSP1.2.

### Systemic humoral immune responses

**Figure 3:**
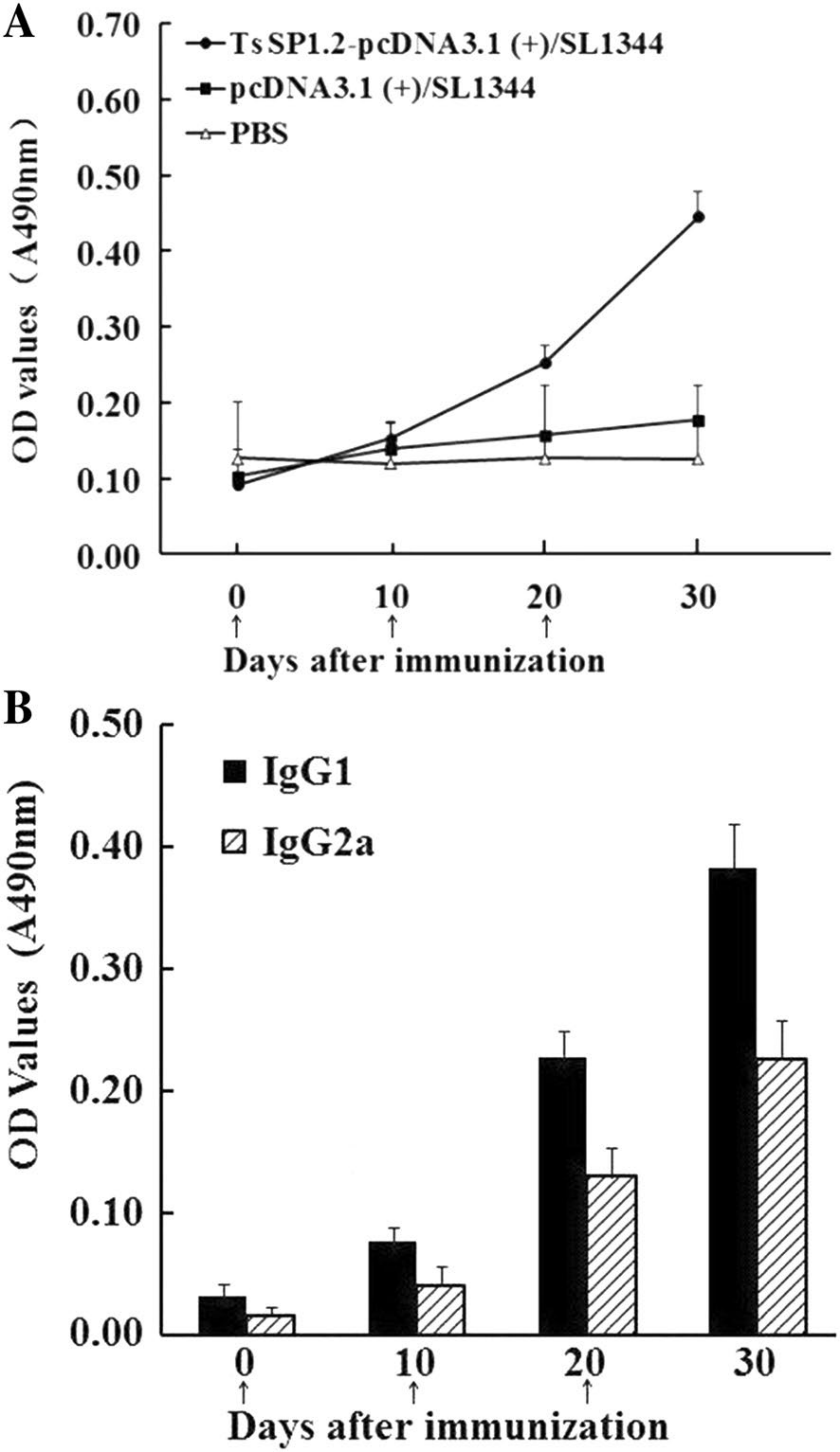
**Serum anti-TsSP1.2 IgG (A) and its subclass (B) responses in vaccinated mice were assayed by ELISA with rTsSP1.2.** The OD values of each group are the mean ± SD of 10 mice. The time point of vaccination is marked as arrows (↑).

Serum anti-TsSP1.2 IgG and its subtype (IgG1 and IgG2a) at 10 days after each immunization were assayed by ELISA. Anti-TsSP1.2 IgG levels in mice vaccinated with TsSP1.2 DNA/*S. typhimurium* was obviously elevated after the 2^nd^ and  3^rd^ vaccination (Figure 3A). But, two groups of mice inoculated with empty pcDNA3.1or PBS did not exhibit apparently detectable anti-TsSP1.2 IgG responses. To evaluate the ability of the TsSP1.2 DNA vaccine to elicit Th1 or Th2-like responses, serum level of anti-TsSP1.2 IgG subclass was also determined. The level of anti-TsSP1.2 IgG1 and IgG2a was also elevated evidently in mice vaccinated with TsSP1.2 DNA/*S. typhimurium* after the 2^nd^  and 3^rd^ vaccination (Figure 3B). The IgG1 level on day 20 and 30 following the first vaccination was distinctly higher than those of IgG2a (t_20days_ = 10.527, t_30days_ = 10.971, *P* < 0.01). Nonetheless, it is obvious that IgG2a was also triggered after the third vaccination, demonstrating that the concurrent Th1/Th2 immune response was elicited by immunization with TsSP1.2 DNA/*S. typhimurium*.

### Cytokines from TsSP1.2-stimulated spleen and MLN cells of vaccinated mice

**Figure 4:**
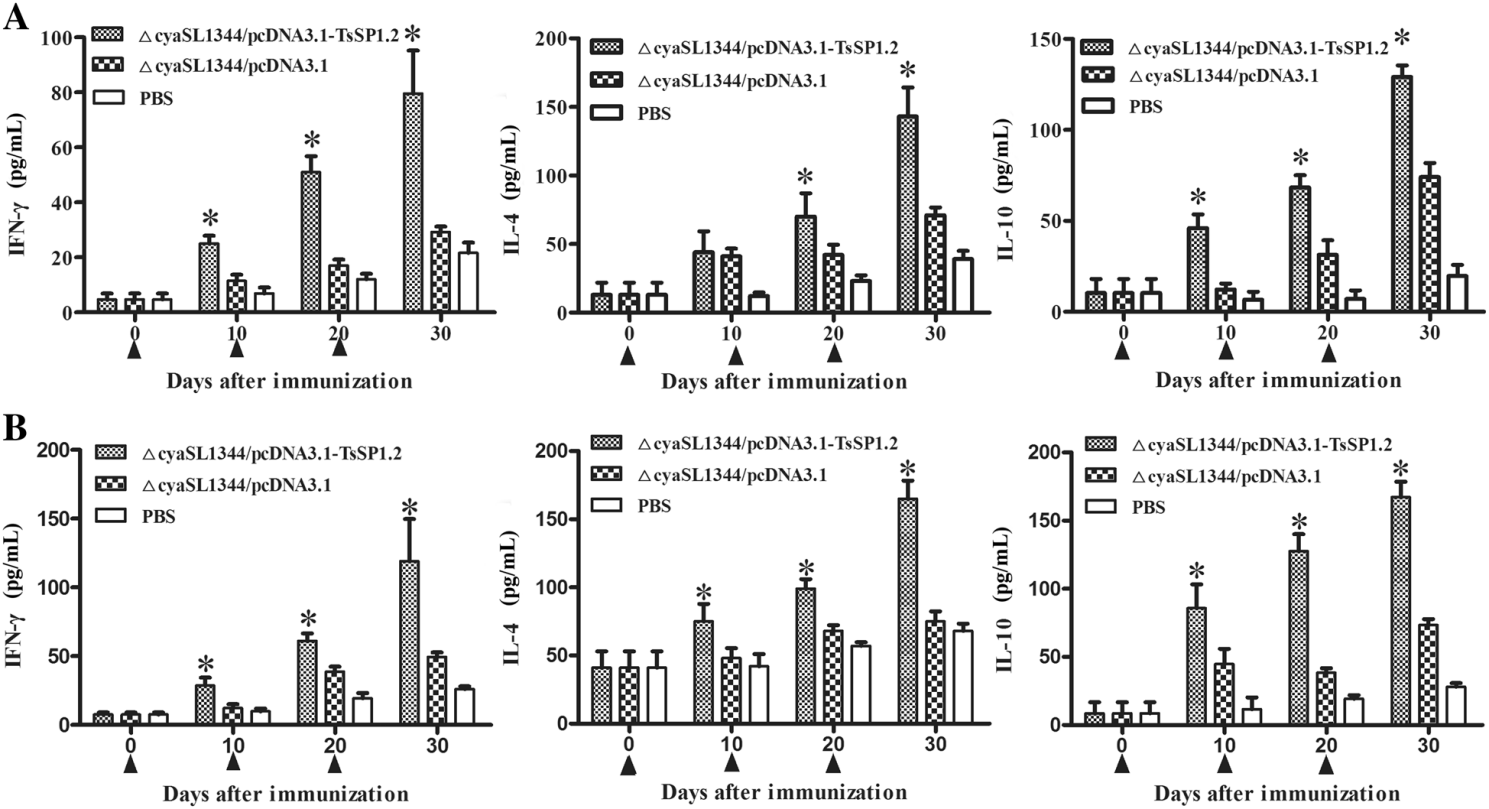
**IFN-γ, IL-4 and IL-10 levels determined in supernatants of splenocytes (A) and MLN cells (B) of mice orally vaccinated with ΔcyaSL1344/pcDNA3.1-TsSP1.2.** After the cells were stimulated with rTsSP1.2 for 72 h, cytokine concentration in supernatants was determined by ELISA. The data are the mean ± SD of cytokine concentration (*n* = 5). Asterisk (*) indicates statistical differences (*P* < 0.01) compared with two control groups.

To evaluate the cytokine production induced by vaccination with ΔcyaSL1344/pcDNA3.1-TsSP1.2, spleen and MLN cells were collected from three groups of mice and stimulated with rTsSP1.2. Cytokines in supernatants were quantified by ELISA. At day 10 after immunization, significant higher levels of IFN-γ and IL-10 were observed in spleen and MLN cells from immunized mice with respect to empty plasmid pcDNA3.1 and PBS groups (*P* < 0.01) (Figure 4). At days 20 and 30 after vaccination, levels of IFN-γ, IL-4 and IL-10 in spleen and MLN cells of immunized mice were evidently elevated relevant to the plasmid alone and PBS groups (*P* < 0.01), demonstrating that oral vaccination of ΔcyaSL1344/pcDNA3.1-TsSP1.2 elicited the concurrent Th1/Th2 immune responses.

### Intestinal mucosal immune response

**Figure 5:**
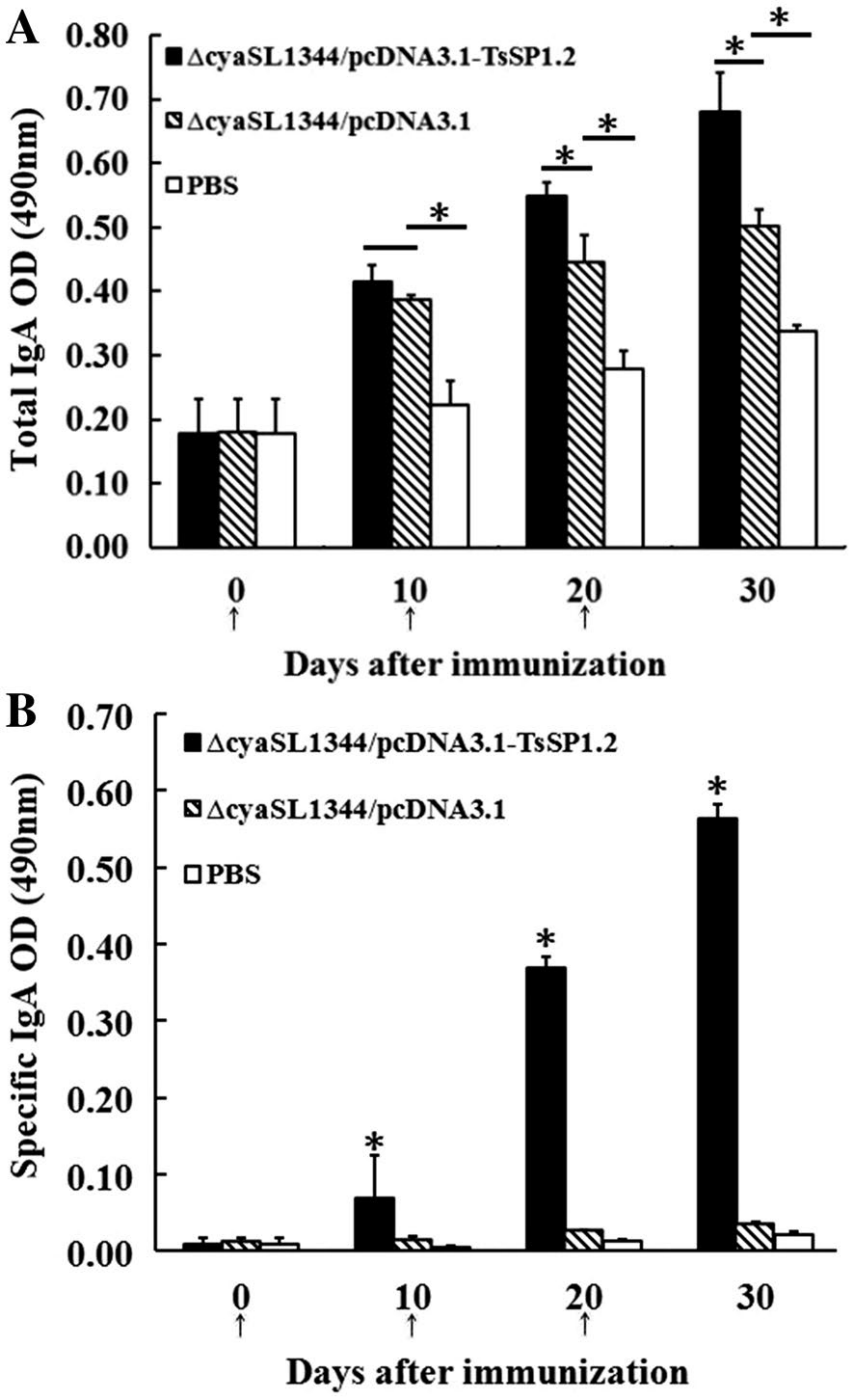
**Total IgA (A) and specific anti-TsSP1.2 IgA (B) in intestinal lavages of mice vaccinated with TsSP1.2 DNA vaccine, plasmid alone or PBS.** Data are the mean ± SD of five mice per group. Asterisks (*) indicate statistically significant differences (*P *< 0.01) relative to empty plasmid alone or PBS groups.

Total intestinal mucosal sIgA was assayed by a sandwich ELISA. TsSP1.2-specific sIgA was assayed by an indirect ELISA using rTsSP1.2-coated plates. Total intestinal sIgA level was obviously increased in mice vaccinated with ΔcyaSL1344/pcDNA3.1-TsSP1.2 and empty plasmid relative to those inoculated with only PBS (Figure 5A) (*F* = 93.815, *P* < 0.01). Specific anti-TsSP1.2 sIgA was evidently elevated in mice vaccinated with TsSP1.2 DNA vaccine with respect to those inoculated with plasmid alone or only PBS (t_plasmid_ = 54.551, t_PBS_ = 63.619, *P* < 0.01) (Figure 5B). No specific mucosal sIgA was detected in mice inoculated with empty plasmid or PBS only.

### Recognition of the native TsSP1.2 at different *T. spiralis* phases

**Figure 6:**
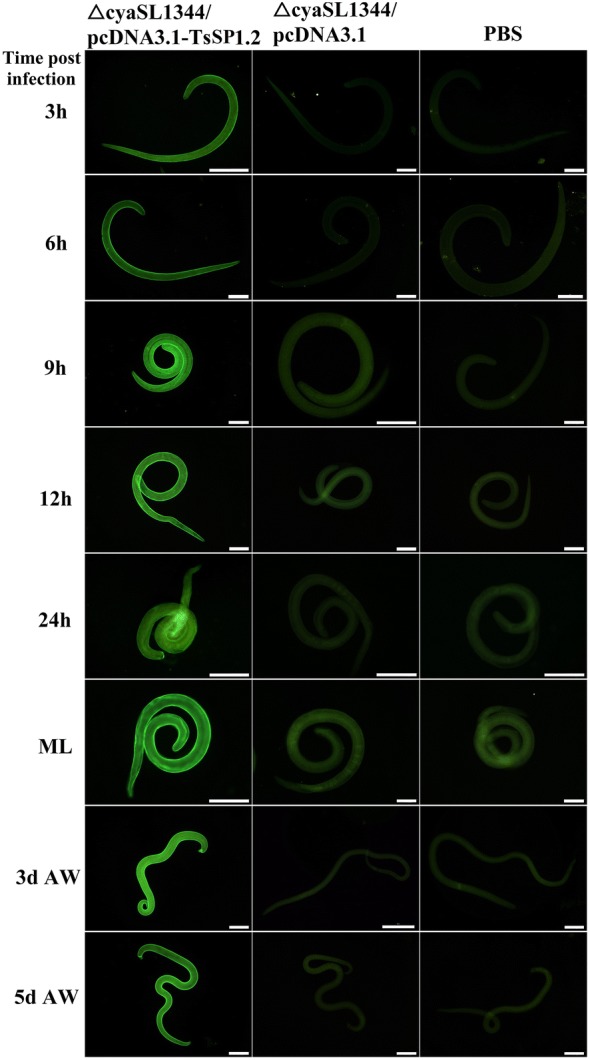
**Recognition of native TsSP1.2 on the surface of various** ***T. spiralis*** **phases by IFT with serum of mice immunized with** ***Salmonella*** **ΔcyaSL1344/pcDNA3.1-TsSP1.2, plasmid alone or PBS.** Scale bar = 100 μm.

**Figure 7:**
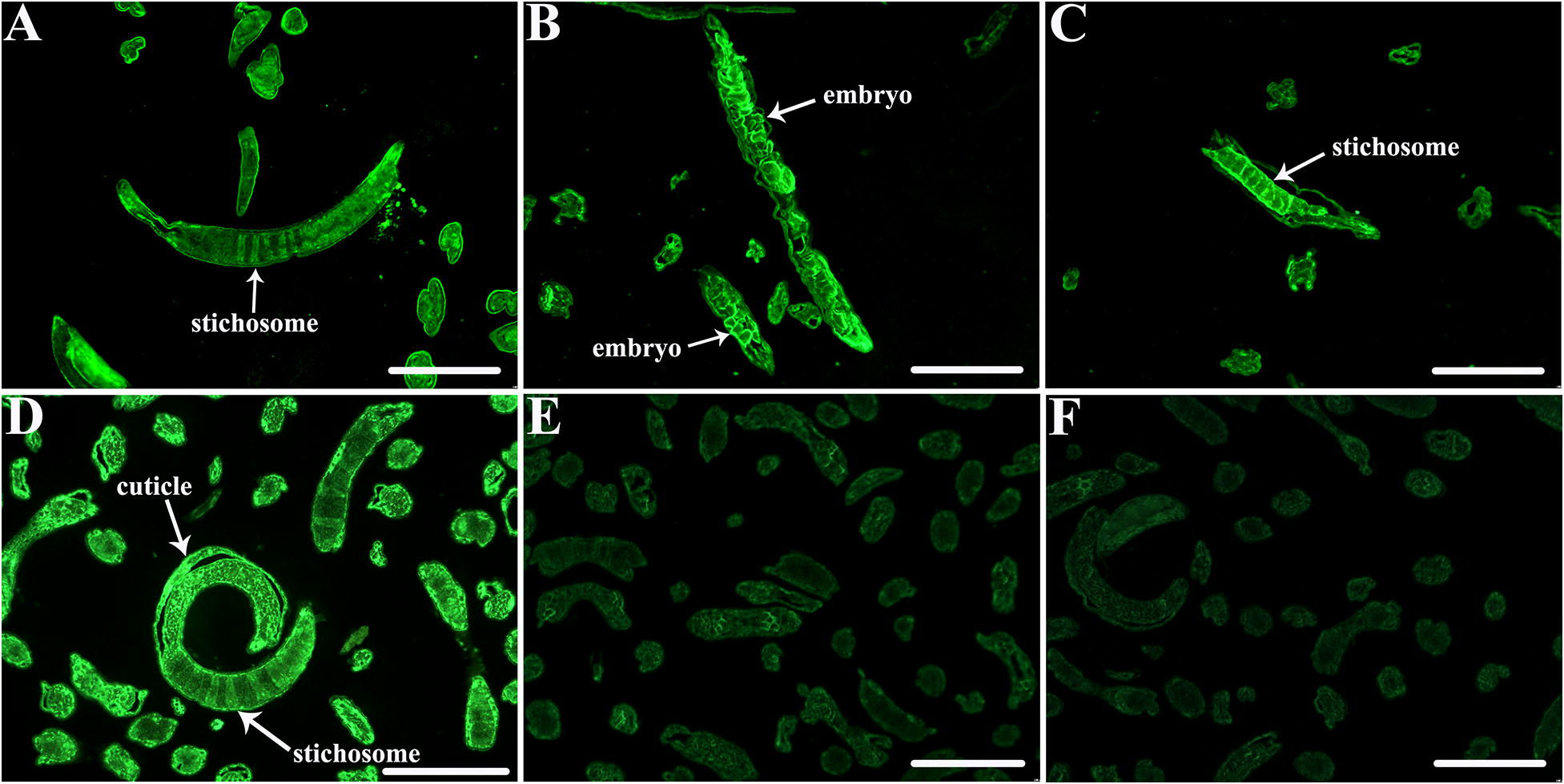
**Immunolocalization of TsSP1.2 at** ***T***. ***spiralis*** **various phases by IFT with serum of mice vaccinated with** ***Salmonella*** **ΔcyaSL1344/pcDNA3.1-TsSP1.2, plasmid alone or PBS.** The staining was located at the cuticle and stichosome of IIL (**A**), adult females (**B**, **C**), ML (**D**), and embryos in adult females (**B**). ML reveals no immunostaining with serum of mice inoculated with plasmid alone (**E**) or PBS (**F**) as negative control. Scale-bar = 100 μm.

IFT analysis revealed that anti-rTsSP1.2 serum recognized the native TsSP1.2 on worm surface, fluorescent sign was homogenously distributed along the cuticle of various stages of *T. spiralis* (e.g., 3–24 h IIL, 3 and 5 day AW and ML) (Figure 6). When parasite sections were probed by anti-rTsSP1.2 serum, immunostaining was located at the cuticle and stichosome of IIL, AW, ML and embryos (Figure 7). Serum from mice inoculated with empty plasmid alone or PBS did not recognize any surface or internal components of the nematode. When intestinal anti-TsSP1.2 sIgA from immunized mice was used, the native TsSP1.2 was also detected on the surface of various stages of this nematode (Additional file 1). However, *T. spiralis* cuticles were not recognized by intestinal washings from mice inoculated with only empty plasmid or PBS.

### Immune protection against *T. spiralis* larval challenge

**Figure 8:**
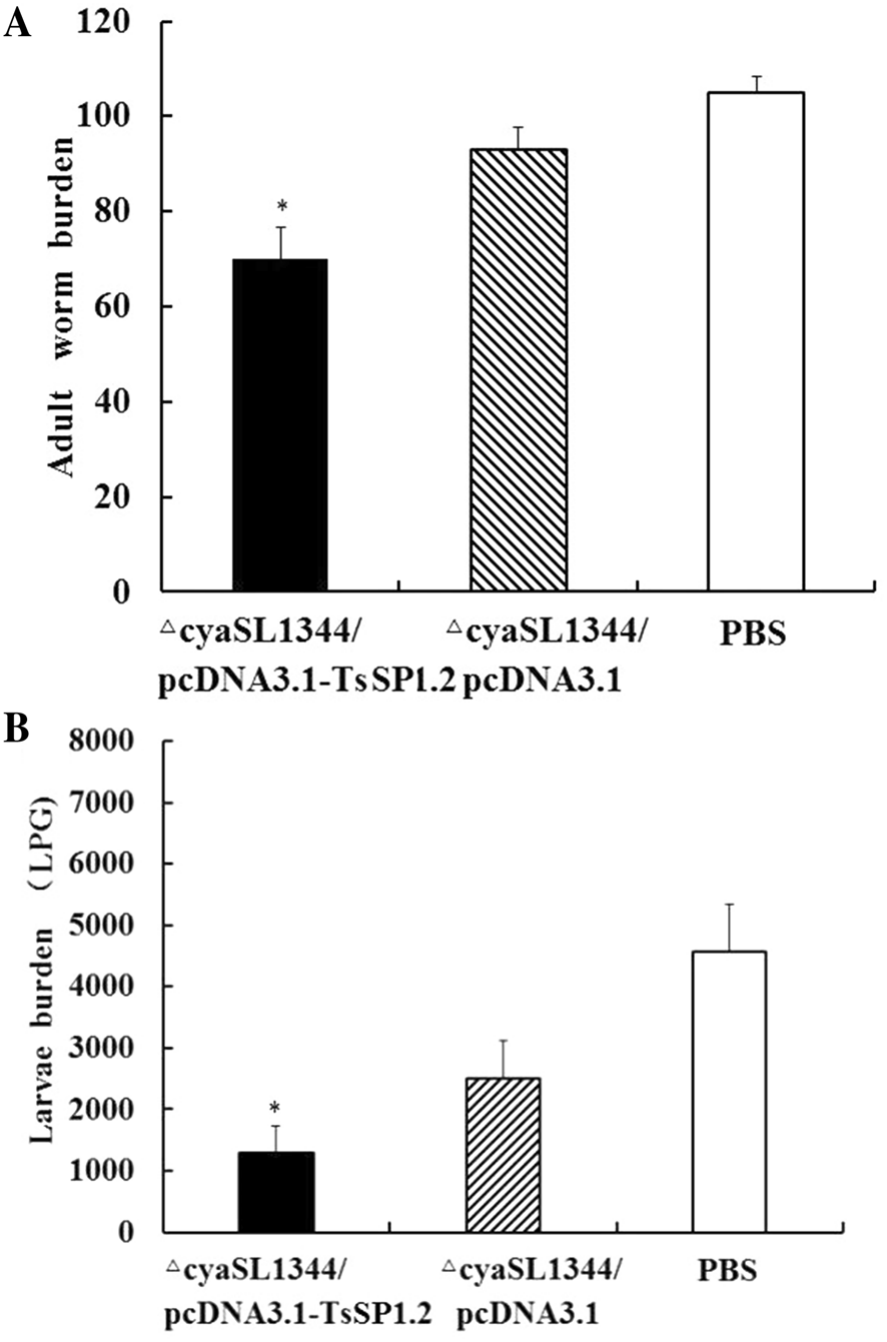
**Immune protection of mice vaccinated with ∆cyaSL1344/pcDNA3.1-TsSP1.2 after being challenged with 300** ***T. spiralis*** **larvae. A** Intestinal adult worm number. **B** Larvae per gram (LPG) of muscles. Data are shown as the mean ± SD of 10 mice/group. A statistically distinct reduction was observed in the parasite burdens of intestinal adults and muscle larvae in immunized mice compared to plasmid alone or PBS group (*P < 0.01).

The immune protection was investigated in vaccinated mice against challenge infection with *T. spiralis* ML. The results revealed that the mice vaccinated orally with ΔcyaSL1344/pcDNA3.1-TsSP1.2 produced a 33.45% reduction in intestinal adult recovery and 71.84% reduction in muscle larval recovery (Figure 8), compared to PBS control mice (F_adults_ = 94.854, F_larvae_ = 69.003, *P* = 0). Additionally, the difference of adult burden (t = 7.842, *P* = 0) and muscle larval burden (*t* = 5.057, *P* = 0) between TsSP1.2 DNA vaccine and the empty plasmid group was statistically significant. The results indicate that a significant immune protection against *T. spiralis* challenge was elicited by oral vaccination of mice with TsSP1.2 DNA vaccine.

**Figure 9:**
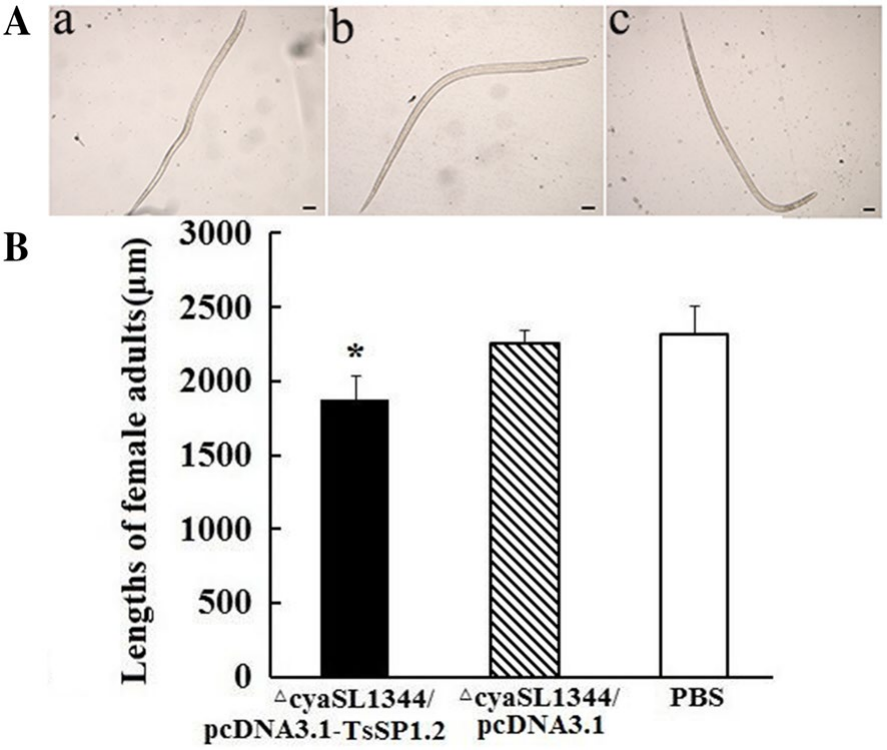
***T. spiralis*** **adult females collected from mice vaccinated with ∆cyaSL1344/pcDNA3.1-TsSP1.2 at 7** **days after challenge. A** Morphology of *T. spiralis* adult females (Scale bar = 100 μm) from mice vaccinated with Δ*cya*SL1344/pcDNA3.1-TsSP1.2 (a), Δ*cya*SL1344/pcDNA3.1 alone (b) and only PBS (c) groups. **B** The mean length ± SD of 10 adult females from each group. Asterisk demonstrates an evident differences (*P *< 0.01) in female length relative to the two control groups.

**Figure 10:**
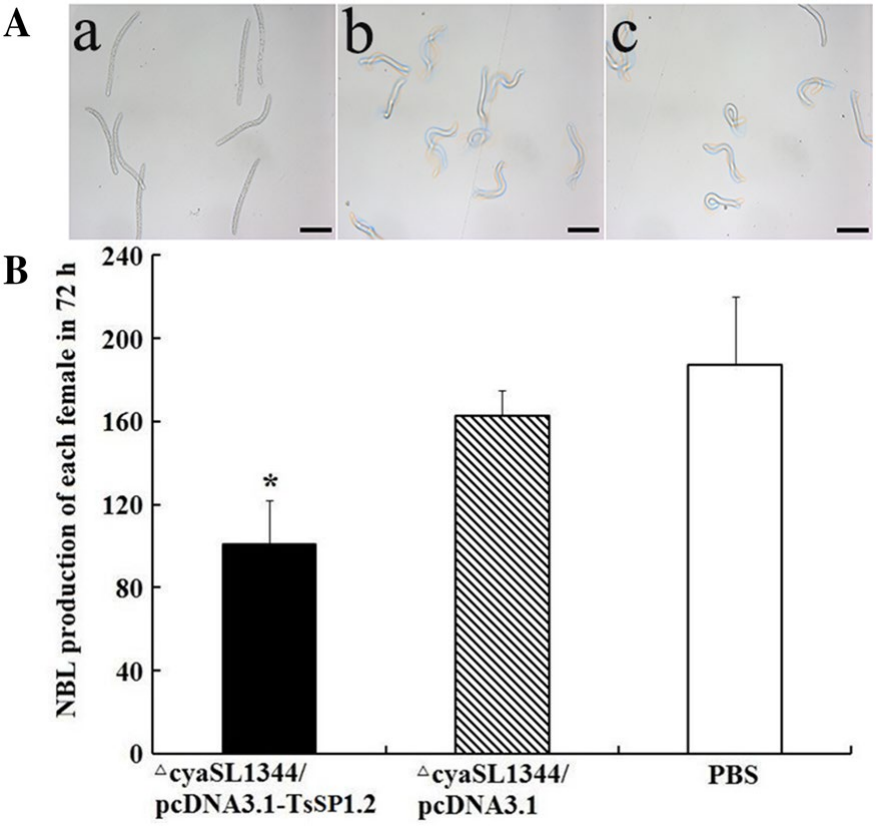
**The in vitro newborn larvae produced by adult females from mice vaccinated with ∆cyaSL1344/pcDNA3.1-TsSP1.2 after challenge. A** Morphology of *T. spiralis* newborn larvae deposited by adult females collected from mice vaccinated with ΔcyaSL1344/pcDNA3.1-TsSP1.2 (a), ΔcyaSL1344/pcDNA3.1 (b), and PBS alone (c). **B** The mean ± SD of the in vitro newborn larvae production in 72 h of 10 adult females. Asterisk indicates statistically differences *(P* < 0.01) in newborn larvae production with respect to both control groups. Scale bar = 50 μm.

Furthermore, intestinal adult females collected from mice immunized with TsSP1.2 DNA vaccine were significantly smaller than those from mice inoculated only by plasmid or PBS control (t_plasmid_ = 6.285; t_PBS_ = 5.551, *P* = 0) (Figure 9). The in vitro NBL production of adult females from mice immunized with TsSP1.2 vaccine was evidently lower than those from mice inoculated with plasmid alone or PBS control (t_plasmid_ = 5.311; t_PBS_ = 4.986, *P* = 0.001). Moreover, the NBL activity from the TsSP1.2 vaccine group becomes weaker with respect to two controls (Figure 10). The results indicate that larval growth and development and female fecundity were inhibited in immunized mice.

## Discussion

Serine protease is one important family of proteases and plays a significant function in parasite infection [40, 41]. The proteolytic enzyme is involved in larval invasion, molting, and development [42]. *Trichuris muris* serine proteases degraded intestinal mucin Muc2 and destroy the mucus barrier [43]. Some *T. spiralis* serine proteases have been identified and characterized in ES proteins of ML, IIL and AW [16, 44, 45]. While the IIL invaded IEC, the expression level of IIL serine protease was significantly increased in comparison with the ML [46]. Monoclonal antibodies against *T. spiralis* TsSP1 inhibited the parasite invasion of IEC in vitro, indicating that TsSP might exert a principal function for degrading intestinal epithelial proteins and promoting larval invasion [47]. Subcutaneous vaccination of mice with rTsSP1.2 protein induced a 34.92% and 52.24% worm reduction of intestinal AW and muscle larvae, respectively [18]. The mice vaccinated with recombinant serine protease rTs-Adsp from *T. spiralis* adults showed a 46.5% ML reduction [48]. Immunization of mice by intramuscular injection of DNA vaccine from *T. spiralis* NBL serine protease exhibited a 77.93% larval reduction post challenge [39]. The results demonstrate that *T. spiralis* serine proteases participated in larval invasion of intestinal mucosa and could be deemed as anti-*Trichinella* vaccine target molecules.

Since trichinellosis is mainly due to the ingestion of infected meat, oral immunization is a more appropriate route to induce long intestinal protective immunity [13, 49]. Previous studies showed that attenuated *Salmonella* is an efficient live carrier for antigen delivery to elicit permanently persistent systemic and mucosal protective responses to the intestinal parasite stages, providing a valid vaccination strategy by means of oral or intranasal routes [50, 51]. Intestinal mucosal IgA response and secretory IgA (sIgA) cells returning to intestinal epithelium could be effectively elicited by oral vaccination of the attenuated *Salmonella*, the sIgA has crucial roles for expelling or sacrificing intestinal parasites [52]. An oral Ts87 DNA/*S. typhimurium* vaccine showed protection against *T. spiralis* challenge as demonstrated by a 29.8% and 34.2% worm reduction of AW and ML in a mouse model [26]. Oral vaccination of mice with *T. spiralis* paramyosin (TsPmy) DNA/*S. typhimurium* induces a 44.8% and 46.6% reduction of AW and ML in vaccinated mice [30]. Intranasal immunization of mice with attenuated *Salmonella* carrying *T. spiralis* gp43 antigen-derived 30-mer epitope (Ag30) produces a 61.83% reduction of adult burden at 8 dpi; when the attenuated *Salmonella* vaccine that secretes Ag30 combined with adjuvant C3d-P28 was used, the adult reduction was up to 92.8% following challenge [31, 33].

In our study, to enhance the efficacy of TsSP1.2 vaccination, oral vaccination with *T. spiralis* TsSP1.2 DNA delivered through attenuated *S. typhimurium* shows a significant immune protection against challenge in vaccinated mice. This protection exhibited obvious reductions of intestinal adult and muscle larval burdens. The attenuated *Salmonella* is a live vector in which the target DNA can be carried to local and systemic lymph tissues [53]. The TsSP1.2 mRNA and rTsSP1.2 protein in spleens and MLN of immunized mice were detected by RT-PCR and IFT, indicating that the TsSP1.2 gene was transcribed and expressed in mice after vaccination with TsSP1.2 DNA/*S. typhimurium* vaccine. Oral vaccination with attenuated *Salmonella*-delivered TsSP1.2 DNA trigged evidently not only local intestinal mucosal sIgA responses but also systemic immune responses. The sIgA exerts a key function in intestinal defense and blocking of parasite invasion of intestinal epithelium. The sIgA against adult surface antigens mediated intestinal adult worm expulsion, passive transfer of McAb IgA against *Trichinella* to naive mice produced 95% protection against larval challenge infection [54, 55]. Besides, the protection might be due to the formation of anti-*T. spiralis* antibody immune complex in the larval head, which may physically block larval direct contact with IEC, therefore protecting intestinal mucosa from larval invasion [18, 56, 57]. Our results reveal that immunized mice produced the TsSP1.2-specific intestinal sIgA, and serum IgG, which recognized the native TsSP1.2 on the surface or secreted by various *T. spiralis* stages. The sIgA is Th2-dependent, especially IL-4 and IL-10 are the main cytokines which strengthen sIgA responses [58], suggesting that IL-4 as well as IL-10 may also enhance intestinal sIgA response.

Additionally, intestinal sIgA could also inhibit the female worm fecundity, reduce adult size and block larval establishment in intramulticellular niches of intestinal mucosal columnar epithelium [33, 59]. Our results reveal that the length of adult females recovered from immunized mice and their fecundity (the in vitro NBL production of females in 72 h) were significantly lower than those from empty plasmid alone or PBS groups. The results suggest that TsSP1.2-specific intestinal sIgA inhibited the intestinal worm growth and reduced female fecundity, since female uterus length is related to reproductive capacity index, i.e., the shorter the uterus, the lower the intrauterine larval capacity and reproductive capacity index [60, 61]. TsSP1.2 might be a pivotal protease related to the larval invasion of the host’s intestinal mucosa. The high levels of TsSP1.2-specific IgG and sIgA produced by oral vaccination with rTsSP1.2 might inhibit larval invasion and development, and reduce female fecundity [57, 59, 62]. Furthermore, anti-TsSP1.2 antibodies might take part in the killing of newborn larvae by an ADCC-mediated mechanism [63]. Therefore, oral vaccination with TsSP1.2 DNA vaccine produced a significant reduction of muscle larva burden in immunized mice.

Humoral immune responses revealed that serum IgG1 levels were more obviously higher than those of IgG2a on days 20 and 30 after oral vaccination with TsSP1.2 DNA, but IgG2a was also elicited following vaccination, suggesting that the mixed Th1/Th2 immune response was triggered with immunization with TsSP1.2 DNA/*S. typhimurium*. The mixed immune response was further verified by higher levels of Th1 (IFN-γ) and Th2 cytokines (IL-4 and IL-10) after spleen and MLN cells of immunized mice were stimulated by rTsSP1.2 protein. The mixed Th1/Th2 immune response is important for immune protection against larval challenge infection [33, 64]. Helminth infections are usually associated with Th2-type immune response characterized by the activation of CD4+ T helper cells and secretion of IL-4, IL-5, IL-9 and IL-13. The Th2-type cytokines are important for intestinal *T. spiralis* adult worm expulsion, which is regulated mainly by Th2-type cytokines and depends on IL-4/IL-13 production; when they are inhibited, the parasite survival is extended [13, 65]. Additionally, Th2-type cytokines also activate macrophages that mediate the host defense against the nematode infection.

Since *T. spiralis* is a multicellular parasitic nematode with complicated antigenicity, the immune responses elicited by vaccination with a single recombinant protein molecule might not be enough to confront challenge infection [13]. Vaccination of mice with TsSP1.2 DNA/*S. typhimurium* produced a partial immune protection. Consequently, to improve the protective efficacy, oral polyvalent vaccines against different *T. spiralis* invasive stages need to be further exploited [9, 33, 39, 66]. Additionally, different adjuvants may elicit different immune protection, the adjuvants (e.g., Montanide, nanoparticles or Th2 cytokine) and other administration means (intranasal route) should also be attempted to induce better immune protection with rTsSP1.2 [67, 68].

In conclusion, our results demonstrate that a concurrent Th1/Th2 immune response and intestinal mucosal IgA response were elicited by oral vaccination with attenuated *Salmonella*-delivered TsSP1.2 DNA vaccine. The immunized mice show an evident immune protection against *T. spiralis* challenge as demonstrated by a 33.45% reduction of intestinal adult worms and 71.84% reduction of muscle larvae. The protection might be due to rTsSP1.2-induced production of specific anti-TsSP1.2 sIgA, IgG, IgG1/IgG2a, and secretion of IFN-γ, IL-4 and IL-10, which could protect intestinal mucosa from the parasite invasion, inhibit worm development and reduce female fecundity. The attenuated *Salmonella*-delivered rTsSP1.2 DNA offers a prospective strategy for the prevention and control of animal *Trichinella* infection.

## Additional file

**Additional file 1.** **Recognition of the native TsSP1.2 on surface of different** ***T. spiralis*** **phases by IFT with intestinal washes from mice vaccinated with ⊿cyaSL1344/pcDNA3.1-TsSP1.2, empty plasmid or PBS.** Scale bar = 50 μm.

## Abbreviations

AW: adult worms
BHK: baby hamster kidney cells
dpi: days post-infection
ES: excretory–secretory
IEC: intestinal epithelial cell
IFT: immunofluorescent test
IIL: intestinal infective larvae
LPG: larvae per gram
MBP: maltose-binding protein
ML: muscle larvae
MLN: mesenteric lymph nodes
NBL: newborn larvae
OPD: *o*-phenylenediamine dihydrochloride
ORF: open reading frame
SD: standard deviation
sIgA: secretory IgA
SPF: specific pathogen-free
TsSP: *T. spiralis* serine protease
