# Supplementary figures and images for: Protective immunity against Trichinella spiralis in mice elicited by oral vaccination with attenuated Salmonella-delivered TsSP1.2 DNA
Source: Vet Res. 2018 Sep 6;49:87. doi: 10.1186/s13567-018-0582-2 (PMC6127904; doi:10.1186/s13567-018-0582-2)

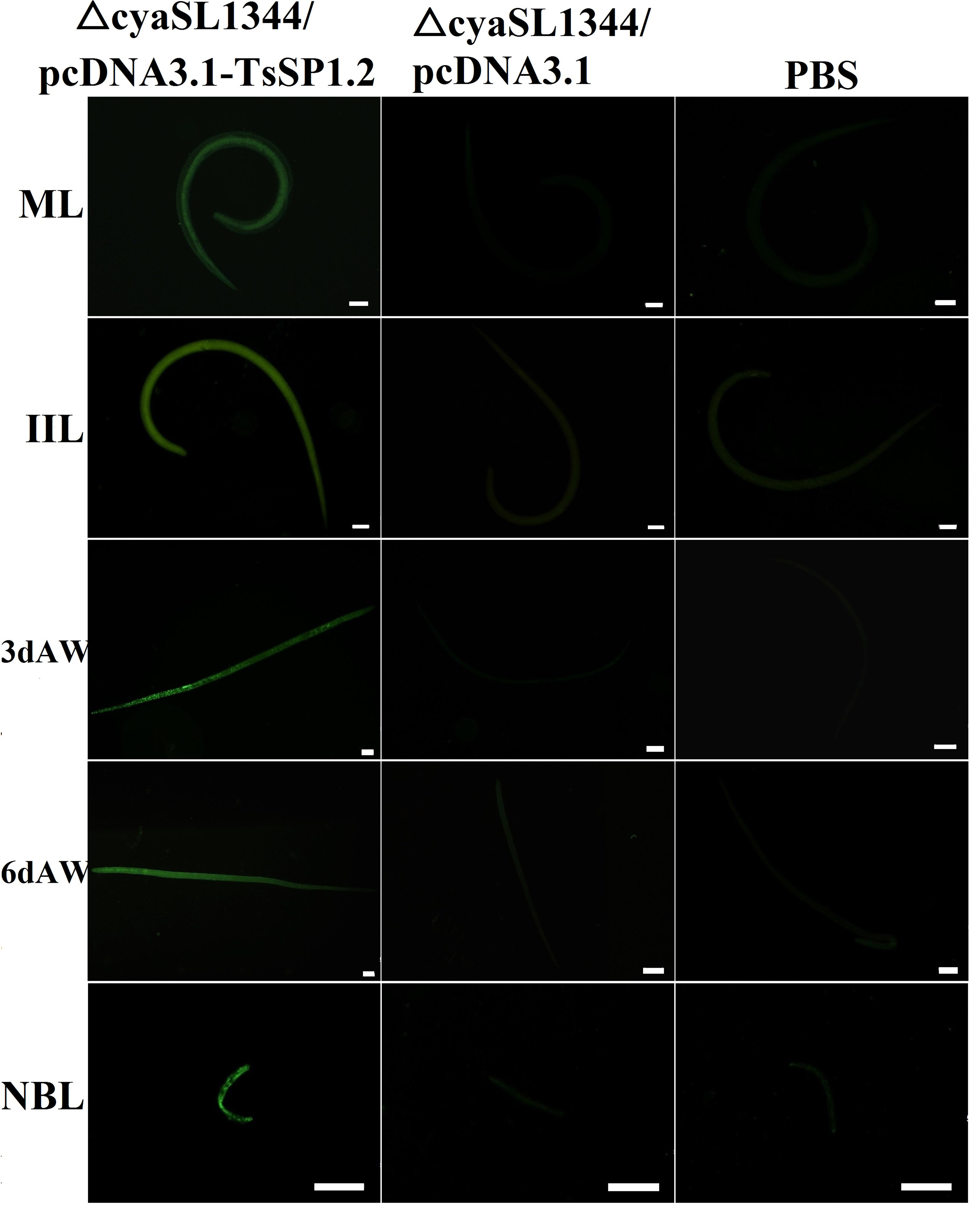

Supplement: Supplementary file 1 — Additional file 1. Recognition of the native TsSP1.2 on surface of different T. spiralis phases by IFT with intestinal washes from mice vaccinated with ⊿cyaSL1344/pcDNA3.1-TsSP1.2, empty plasmid or PBS. Scale bar = 50 μm. [file 13567_2018_582_MOESM1_ESM.jpg]
